# Novel amphiphilic folic acid-cholesterol-chitosan micelles for paclitaxel delivery

**DOI:** 10.18632/oncotarget.13757

**Published:** 2016-12-01

**Authors:** Li-Chun Cheng, Yan Jiang, Yu Xie, Lu-Lu Qiu, Qing Yang, Hui-Yi Lu

**Affiliations:** ^1^ Department of Pharmacy, The Second Affiliated Hospital of Dalian Medical University, Liaoning, Dalian 116027, China; ^2^ Department of Anesthesia, Critical Care and Pain Medicine, Massachusetts General Hospital, Boston, MA 02144, USA

**Keywords:** paclitaxel, folate acid, cholesterol, chitosan, micelle

## Abstract

In order to decrease the toxicity of paclitaxel (PTX) and increase the efficiency, we developed an amphiphilic PTX injection system using a biodegradable and biocompatible polymer synthesized by folic acid, cholesterol, and chitosan (FACC). This FACC-based polymer had a low critical concentration (64.13μg/ml) and could self-assemble in aqueous condition to form nanoscale micelles. The particle sizes of FACC-PTX micelles were 253.2±0.56 nm, the encapsulation efficiency and loading capacity of these FACC-PTX micelles were 65.1±0.23% and 9.1±0.16%, respectively. The cumulative release rate was about 85% at pH 5.0 which was higher than that at pH 7.4 (76%). This pH-dependent release behavior was highly suggesting that PTX release from FACC-PTX micelles might be higher in a weak acidic tumor microenvironment and lower toxic for normal cells. The anti-cancer effectiveness of FACC-PTX micelles was investigated by *in vitro* cytotoxicity and targeting study. The results revealed that FACC micelles have non-toxic on cells as evidenced by high cell viability found (86% to 100%) in the cells cultured with various concentrations of FACC micelles (1 to 500 μg/ml). Targeting study indicated that the cytotoxic efficacy of FACC-PTX micelles was significantly higher than that with Taxol® in the Hela cells (folate receptor-positive cells). These findings indicated that the anticancer efficiency of PTX can be enhanced by adding some cancer cell positive receptor into drug carrier and the FACC micelle was a potential tumor targeting carrier for PXT delivery.

## INTRODUCTION

Paclitaxel (PTX) is an effective and wide spectrum chemotherapeutic agent, which has been successfully used in the clinical treatment of many solid tumors, especially breast and ovarian tumor [1]. However, the poor solubility and high toxicity of PTX limits the medical applications of this drug. In order to realize the targeted delivery of PTX to tumor, many methods have been studied. One of successful medications was a commercial drug named Taxol® formulated with Cremophor EL® (polyethoxylated castor oil) and dehydrated ethanol (50:50 v/v). However, cremophor EL® can induce severe side effects as hypersensitivity, neurotoxicity, and nephrotoxicity [2]. The other studies have largely improved the solubility and side effects of PTX but still remained many limitations. The layer-by-layer assembly of chitosan-PTX liposomes enhanced PTX induced cytotoxicity in human cervical cancer cells as compared to PTX [3]. However, particle flocculation or aggregation was difficult to overcome [4]. Peptide-conjugated biodegradable PTX nanoparticles exhibited significantly stronger antiangiogenic activity than Taxol® on endothelial cells [5]. However, the problems with peptide stability and their short blood half-lives due to peptidase sensitivity would limit the effectiveness of peptide-conjugated drugs. Chitosan-PLGA particles were significantly enhanced PTX cytotoxicity for 4T1 cells [6]. Because carboxylic acid chain ends are the products of the hydrolytic cleavage of PLGA polymers, the degradation of the PLGA polymer may lead to high acidic and painful products locally [7]. Therefore, a less toxic and better-tolerated delivery system for PTX to substitute Taxol® is still a big challenge for both clinical medical doctors and anti-cancer researchers.

Among these new delivery systems, polymeric micelles are recognized as one of the most potential chemotherapeutic agent delivery systems, due to their good solubilization efficiency and reducing non-selective reticuloendothelial system (RES) scavenge. Moreover, the nanoscale dimension polymeric micelles exhibit tumor accumulation by enhancing permeability and retention (EPR) effect [8]. Chitosan is widely distributed in nature and used in micelle system owing to its good physicochemical characters, including biodegradability, biocompatibility, low toxicity, pH sensitiveness and ease of chemical modification [9]. However, chitosan suffers from a poor solubility in water, which is a major drawback for drug formulations [10]. In order to improve the properties of chitosan, chemical modification of the chitosan chains has been investigated. Amphiphilic copolymers present a double affinity for both hydrophilic and hydrophobic environments and are able to self-organize in water to form, in most cases, specific architectures such as micelles or vesicles [11].

In recent years, several chitosan PTX micelle were studied, such as N-succinyl-palmitoyl-chitosan PTX micelles [12], N-octyl-N-(2-carboxylbenzoyl) chitosan PTX micelles [13], amphiphilic carboxymethyl chitosan-quercetin PTX micelles [14] and α-tocopherol succinate-modified chitosan PTX micelles [15]. However, their applications are greatly limited due to high toxicity on normal cells. In order to enhance the tumor targeting of micelles system and decrease the injury to normal tissues, a new modified PTX delivery system is needed. Folic acid (FA) and its derivatives are essential nutrients in humans and play an important role in nucleotide synthesis and methylation reactions. Our hypothesis is that PTX can be delivered effectively by a chitosan modified with folic acid system, which is higher expression in human cancer cells such as Hela and KB cells than in normal cells [16, 17].

The aim of this study is to test our hypothesis by developing a novel amphiphilic folic acid-cholesterol-chitosan-conjugated PTX micelle system to target folate receptor positive cancer cells. Folic acid-cholesterol-chitosan (FACC) is synthesized by aminoacylation reaction of chitosan primary amino groups and FACC-PTX micelles are prepared by dialysis method. Subsequently, the physical properties and biological activity of FACC-PTX micelles are characterized. In addition, cancer targeting specificity of FACC-PTX micelles is determine using Hela (folate receptor-positive) and A549 (folate receptor-negative) cells [16].

## RESULTS

### Synthesis and characterization of CH-CS and FACC

**Figure 1:**
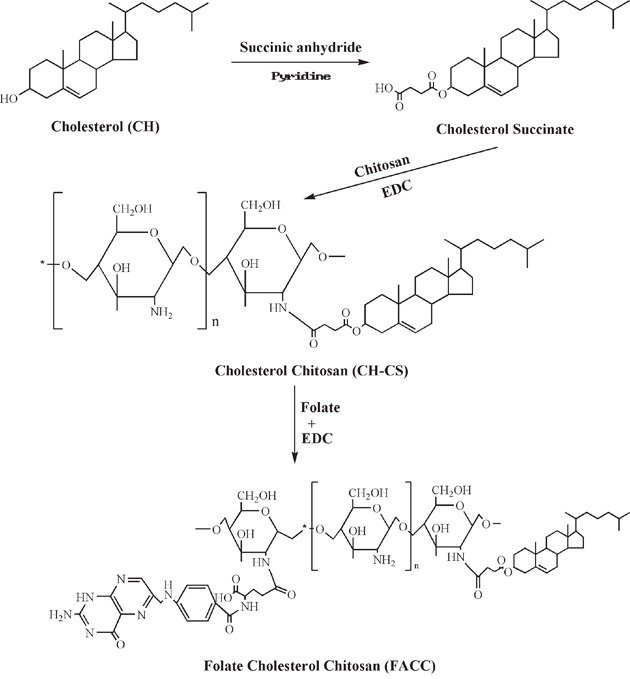
Schematic representation of the synthesis of FACC from cholesterol, chitosan and folate

**Figure 2:**
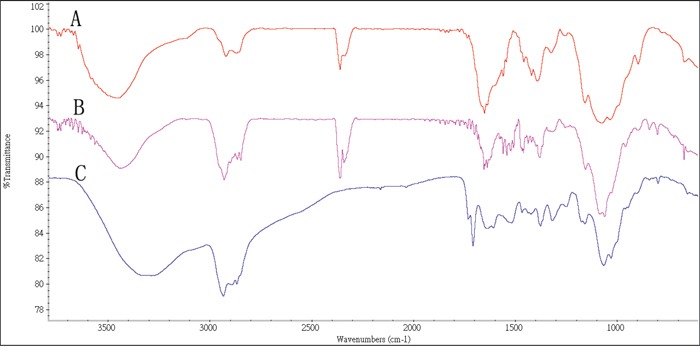
FTIR Spectra of chitosan exhibiting absorbance of -OH and –NH groups at 3468 cm^−1^ A., synthesized cholesterol-chitosan (CH-CS) polymer at 1652 cm^−1^ (C=O stretch overlapped with N-H bend; B., and folate-cholesterol-chitosan (FACC) polymer at 1709 cm-1 (benzene ring; red line: FACC; blue line: CS; C., respectively

In order to develop a novel PTX delivery system, we synthesized a chitosan-based new polymer using folic acid (FA), cholesterol (CH) and chitosan (CS) as described in the scheme (Figure 1). FT-IR spectrum has indicated that a FACC polymer has been synthesized by three-step reactions. At the first step, the hydroxyl group of cholesterol was activated by succinic anhydride to form cholesterol succinate. At the second step, cholesterol succinate reacted with chitosan to create a cholesterol-chitosan (CH-CS) polymer. At the third step, FA reacted with CH-CS polymer to form a FACC polymer. As shown in Figure 2A, the basic characteristics of FT-IR bands of chitosan at 3468 cm^−1^ was assigned to the stretching vibration of O-H and N-H. This peak was still expressed in the FT-IR spectrum of CH-CS polymer (Figure 2B). However, new peak appeared at approximately 1652-1940 cm^−1^ which were attributed to the formation of –NHCO– group in CH-CS (Figure 2B) and FACC (Figure 2C) polymers. The appearance of the peak at 1709 cm^−1^ in the spectrum was attributed to benzene ring of the FA, which stand for amide linkage was identified in FA modified CH-CS (Figure 2C).

**Figure 3:**
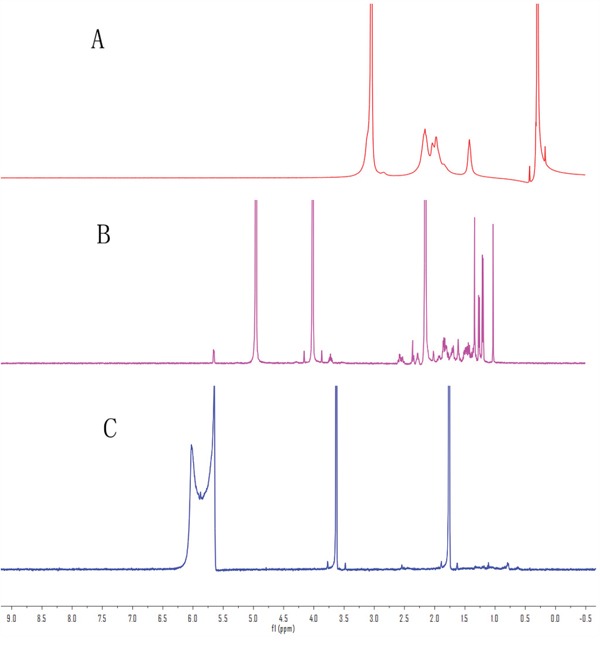
^1^H NMR spectra demonstrating the structure of chitosan A., cholesterol-chitosan B., and folate-cholesterol-chitosan C

^1^H NMR was investigated to further confirm the chemical structure of the CH-CS and FACC. The characteristic peaks of chitosan (Figure 3A) appeared at 1.0-3.0 ppm. Compared with chitosan and CH-CS, new peaks in CH-CS spectrum (Figure 3B) appeared in the range of 3.5-5.5 ppm due to the hydrogen protons of cholesterol moiety, which suggested that cholesterol modified groups were successfully grafted onto the chitosan backbone.

Compared the ^1^H NMR spectrum of FACC (Figure 3C) and CH-CS (Figure 3B), the ^1^H NMR δ spectrum of FACC exhibited the new peaks at 2.5 (br s, β and γ-CH2-groups, 4H), 5.95 ppm (s, aromatic H of pteridine) and 6.65 ppm (benzene ring) as shown in Figure 3. The appearance of these peaks confirmed the successful conjugation of folate with CH-CS.

### Critical micelle concentration (CMC)

**Figure 4:**
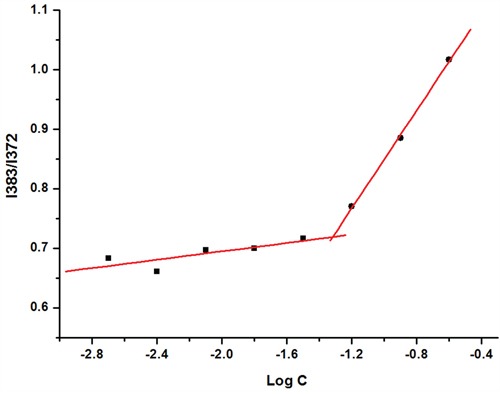
The intensity ratio ((I384/I372) of the pyrene emission spectra versus the log concentrations of FACC The CMC value of FACC was 64.13 μg/ml calculated by the crossover point, which demonstrated that FACC could form very stable micelles at low concentration.

The CMC of the FACC was determined by pyrene fluorescence probe technique. At low concentration (C) of FACC (C<CMC), there were negligible changes in fluorescence intensity ratio of I384/I372. As the concentration increased, remarkable increase of the intensity ratio was observed by fluorescence spectroscopy. The CMC curves of FACC determined from emission spectra of pyrene in FACC solutions were shown in Figure 4. Based on the intensity ratio data, the CMC value of FACC was calculated by the crossover point. The CMC value of FACC was 64.13 μg/ml, which demonstrated that FACC could form very stable micelles at low concentration.

### Physicochemical characterization of PTX-FACC micelles

**Figure 5:**
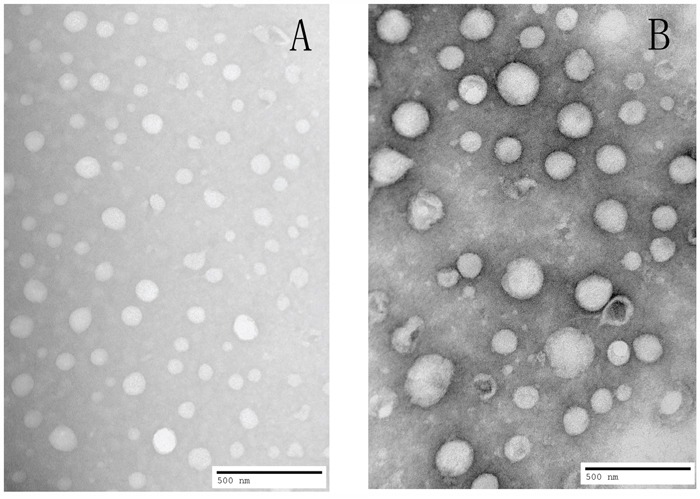
TEM image showing uniform spherical morphology with particle size about 100nm for FACC micelles A. and about 200nm for PTX-FACC micelles B

**Figure 6:**
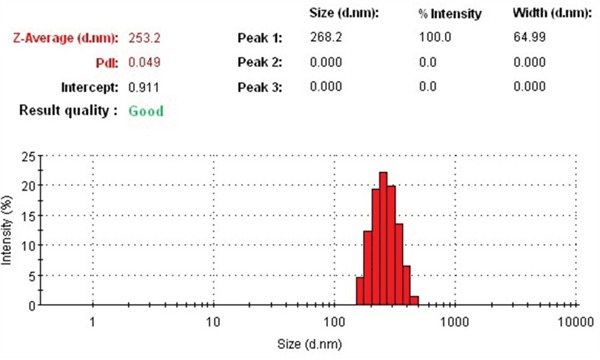
The size distribution of PTX-FACC micelles was in the range of 150-250 nm determined by Zetasizer Nano ZS90 instrument

Dialysis method was used to prepare FACC micelles and PTX-FACC micelles. The encapsulation efficiency (EE), loading efficiency (LE), mean diameter size and polydispersities (PDI) of PTX-FACC micelles were shown in Table 1 with the different dialysis times. The result demonstrated that EE, LE and the micelles size of the micelles were decreased with the dialysis time extension. The PTX-FACC micelles (dialysis time 8 hrs) were both have high EE and LE, but with low particle size, were selected for future research. The morphology of PTX-FACC micelles were determined by TEM (Figure 5). FACC micelles and PTX-FACC micelles had uniform spherical morphology, and the distribution of particle size was in the range of 100-250 nm. These results were in agreement with the measurement of Zetasizer Nano ZS90 instrument (Figure 6).

**Table 1:** Physicochemical characterization of PTX-FACC micelles atdifferent dialysis time points (n=3)

Dialysis time (h)	LE%	EE%	Size (nm)	PDI
4	12.93± 0.67	75.57± 2.14	288.5± 4.78	0.217± 0.073
8	10.52± 1.03	63.13± 3.56	266.0± 1.91	0.107±0.069
12	7.42± 0.87	56.50± 1.96	253.2 ±5.10	0.136± 0.056
24	5.45± 0.62	32.73± 0.98	168.8± 1.036	0.185±0.043

### *In vitro* release of PTX from PTX-FACC micelles

**Figure 7:**
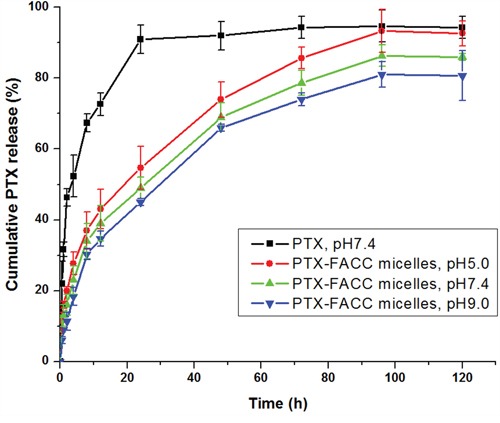
Cumulative release curve of PTX in PBS with 1% polysorbate 80 at different pH conditions Data were given as mean±SD (n = 3).

The concentration of PTX released from PTX-FACC micelles was determined *in vitro* at 37°C in PBS solution with 3 different pH (5.0, 7.4, 9.0) conditions. The PTX released from commercial drug, Taxol was also tested at the same conditions (pH 7.4) and used as a control (PTX group). Our results showed that the releasing process of PTX was faster at acidic condition (pH=5.0) than both physiological condition (pH=7.4) and basic condition (pH=9.0) from FACC-PTX micelles (Figure 7). PTX released from commercial drug, Taxol (PTX group) was much faster than that from FACC-PTX micelles. More than 95% of PTX released from Taxol® at pH 7.4 in 24 hours (Figure 7). However, FACC-PTX micelles released the same levels of PTX at the end of 96 hours. As presented in Figure 7, about 92% of PTX was released within 96 hours at pH 5.0, while about 86% of PTX was released at pH 7.4. This pH-dependent release behavior was highly suggesting that PTX release from FACC-PTX micelles might be higher in a weak acidic tumor microenvironment [18] and lower toxic for normal cells.

### *In vitro* cytotoxicity

**Figure 8:**
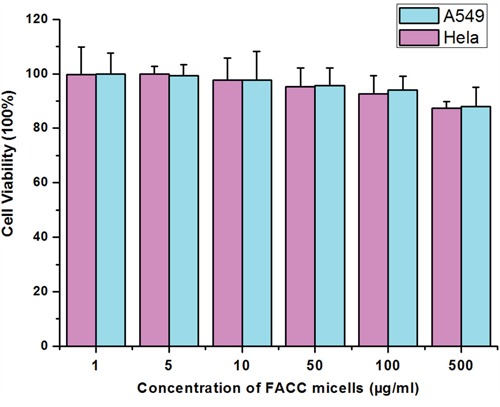
Cytotoxicity of FACC micelles on Hela and A549 cells determined by MTT method As indicated, FACC micelles have non-cytotoxic effect on Hela and A549 cells.

**Figure 9:**
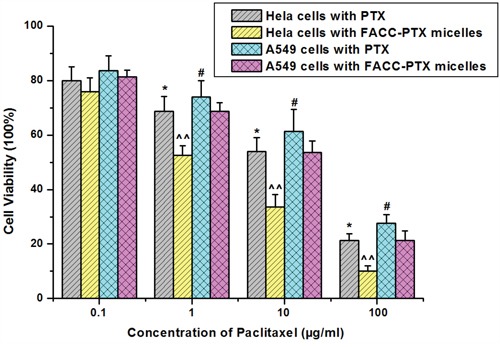
Cytotoxicity of PTX-FACC micelles on Hela and A549 cells determined by MTT method As presented, significantly higher percentages of Hela cells were killed in PTX-FACC micelles-containing medium (PTX-FACC group) compared to those culture with Taxol-containing medium (PTX group) at the same PTX concentrations (* *P*< 0.05). In addition, PTX-FACC had more targeting effect on Hela cells than A549 cells as evidenced by much lower percentages of Hela cells survived than A549 cells in PTX-FACC micelles-containing medium (^^ *P*< 0.01). However, there were no significant differences between Taxol-treated A549 cells and PTX-FACC-treated A549 cells (# *P*> 0.05). Data are presented as mean±SD (*n* = 3).

Cytotoxicity of the FACC-PTX micelles was evaluated using MTT assay on Hela and A549 cells and directly compared their efficacies to that of Taxol®. FACC micelles did not have any obvious effect on the Hela and A549 cells throughout the whole range of concentrations examined (Figure 8). The MTT analyses demonstrated that FACC-PTX micelles were more cytotoxicity than Taxol® (Figure 9). In Hela cells (folate receptor-positive), the cytotoxic efficacy of FACC-PTX micelles was significantly higher than that with Taxol®. However, there was no significantly different cytotoxic efficacy in A549 (folate receptor-negative) cells between FACC-PTX micelles and Taxol®. Besides, in the FACC-PTX micelles groups, the cytotoxicity of Hela cells was significantly higher than that with A549 cells (Figure 9). These results were in accordance with those observed in the FACC-PTX micelles cellular uptake.

### Intracellular uptake of FITC-FACC micelles

**Figure 10:**
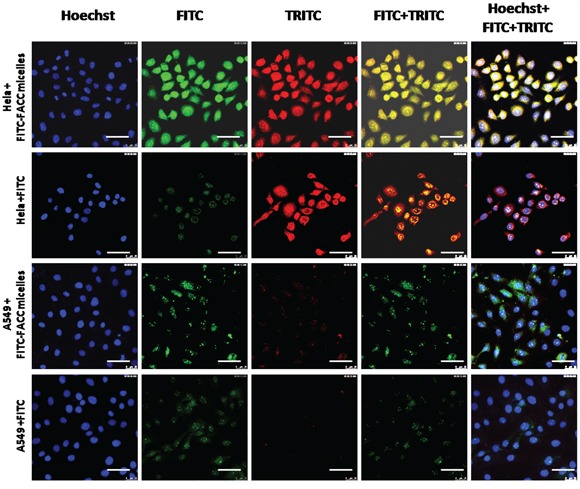
Cellular distribution of FACC micelles in Hela cells and A549 cell for 4 hours, bar=50μm Blue fluorescence from Hoechst labeling the nucleus; green fluorescence from FITC-FACC micelles; red fluorescence from TRITC labeling folate receptor; double fluorescence with FITC and TRITC; treble fluorescence FITC, TRITC and Hoechst. Fluorescence microscope showed that very strong fluorescence signal in FITC-conjugated FACC-PTX treated Hela cells. However, very weak fluorescence signal was found in FITC-conjugated FACC-PTX treated A549 cells. There was no fluorescence signal found in Hela cells and A549 cells treated with FITC solution.

The intracellular uptake efficiency of FITC in Hela cells and A549 cells was investigated using laser confocal microscope. To precisely observe the cellular distributions of FACC micelles, we performed double fluorescence which with green fluorescence from FITC-FACC micelles and red fluorescence from TRITC-folate receptor. The cells were also counterstained with Hoechst 33342 (blue fluorescence) to form treble fluorescence. As shown in Figure 10, mouch higher fluorescence intensity of FITC was found in Hela cells treated with FITC-FACC micelles than that ofHela cells treated with FITC solution and A549 cells treated with FITC-FACC micelles. However, the fluorescence signal neither found in Hela cells nor in A549 cells when they were treated with FITC solution. The fluorescence intensity of TRITC in Hela cells was found to be higher than that in A549 cells, indicating that the Hela cells have express more folate receptor than A549 cells. This observation suggests that the FACC micelles facilitated intracellular uptake of FITC in the positive folate receptor cells.

## DISCUSSION

Nowadays, amphiphilic micelles have been extensively exploited to improve the therapeutic efficiency and reduce severe side effects of anticancer drugs. Chitosan has been chosen as the micelles carrier material because it has many amino groups on the molecular chain and modified easily [19]. Cholesterol-modified O-carboxymethyl chitosan conjugate is amphiphilic in nature and has self-aggregation behavior in aqueous media [20]. Furthermore, chitosan modified with special materials such as folate [21] and RGD [22] polypeptides constitutes active receptor enhanced targeted drug delivery systems. FA is a stable, inexpensive and poorly immunogenic chemical with a high affinity for the FA receptor [23]. Therefore, in this study, FACC was synthesized to enhance the specificity of PTX delivery to tumor cells/tissues.

To prepare low toxic and higher efficacy FACC-PTX micelles, all organic solvents have been removed by dialysis method. Amphiphilic FACC could form to micelles by self-assembly, it was easy to carry hydrophobic anticancer drugs. Folic acid conjugated glycol chitosan micelles for targeted delivery of doxorubicin (DFCHGC) were prepared by emulsion/solvent evaporation method which induced organic solvent ClCH_3_. The size of DFCHGC micelles was from 282 to 320 nm, which was larger than FACC-PTX micelles (150-250 nm) [24]. Hydrotropic oligomer-conjugated glycol chitosan paclitaxel nanoparticles (PTX-HO-CNPs) were prepared by dialysis method with the average size 343 nm, which was also larger than FACC-PTX micelles [2]. Although the loading efficiency of PTX-HO-CNPs were about 96% higher than FACC-PTX micelles (75%), the smaller size of FACC-PTX micelles would batter for tumor targeting by EPR effect.

The FACC has amphiphilic character, which CMC value was 64.13 μg/ml. The higher the degree of substitution of chitosan, the lower the CMC value. The CMCs of N-octyl-N-(2-carboxylbenzoyl) chitosan PTX micelles were from 11 to 72 μg/ml [25]. Cholesterol and folate in the chitosan may have steric hindrance, the degree of substitution of FACC is difficult to be higher. However, our result showed that FACC could form very stable micelles at low concentration.

The result of FACC-PTX micelles *in vitro* release suggested that PTX release rate increased with the decreasing pH value. This result may be attributed to the changes in the structure of the amino of the micelles. In acidic conditions, -NH_2_ in the surface of micelles transformed to –NH_3_^+^ through binding protons. The transition between hydrophilic and hydrophobic groups make micelles unstable under acidic solutions [26]. This pH-dependent release behavior was highly desirable for targeted cancer therapy because it could accelerate the amount of drug released at tumor site and decrease the drug release in normal tissues based on the different acid environment between tumor tissues and normal tissues [27].

Cell viability profile assessed by MTT assay demonstrated that FACC micelles had no significant cytotoxicity at concentration as high as 500 μg/ml, so that FACC micelles may be a safety tool which possibly overcomes anticancer therapy. We also demonstrated that FACC-PTX micelles targeted Hela cells, the cytotoxic efficacy was significant higher than that with A549 cells. Our results indicate that FACC-PTX micelles were a promising nanoscale drug formulation for cancer therapy.

## MATERIALS AND METHODS

### Materials

Paclitaxel (PTX) was purchased from HaiZheng Co. Ltd. (Zhejiang, China); Chitosan (MW 50 kDa, degree of deacetylation >90%), cholesterol, folic acid (FA), 1-(3-dimethylaminoproply)-3-ethylcarbodiimide hydrochloride (EDC), fluorescein isothiocyanate (FITC), phosphate buffered saline (PBS, pH7.4), 3-[4,5-dimethylthiazol-2-yl]-2,5-diphenyltetrazoliumbromide (MTT) and dimethylsulfoxide (DMSO) were purchased from Sigma (St Louise, MO, USA). All solvents and reagents used in this study were analytical grade.

### Cell lines

Human cervices carcinoma cell line (Hela), and human lung adenocarcinoma cell line (A549) were purchased from Blood Research Administration (Tianjin, China). The cells were cultured in Dulbecco Modified Eagle's medium (DMEM) supplemented with 10% fetal bovine serum (FBS), 100 U·mL^−1^ penicillin and 100 μg·mL^−1^ of streptomycin at 37°C with 5% CO_2_.

### Synthesis of cholesterol chitosan (CH-CS)

Cholesterol (0.5 g) and succinic anhydride (0.5 g) were dissolved in 10 ml pyridine. The mixture was stirred for 3h at 70°C and then the solvents were evaporated with vacuum distillation. The precipitate in the reaction mixture was washed three times with ethanol. The white powder of cholesterol succinate was obtained by recrystallization in ethanol. Subsequently, cholesterol succinate (100 mg) was dissolved in 5 ml methanol and activated by addition EDC (50 mg) under stirring for 1h. Then 500 mg chitosan in 50 ml of 1% hydrochloric acid (pH 4.0) was added to the above mixture and the reaction was continued for 24h at 25°C. The resultant mixture was dialyzed against distilled water for 3 days using a dialysis bag (cut-off MWCO 7 kDa). The synthesized product of cholesterol-chitosan (CH-CS) with the molecular weight bigger than 7 kDa was obtained by lyophilization.

### Synthesis of folic acid-cholesterol-chitosan (FACC)

FACC was prepared through aminoacylation reaction (Figure 1). Briefly, 50 mg FA were dissolved in 10 ml anhydrous DMSO with stirring. EDC (3 mM) was added into the solution and stirred at room temperature for 1h. Then 25 mg CH-CS was dissolved in 25 ml of HCl-THF (1% HCl-water: THF = 1/1, v/v) in a new flask to make a clear solution which was added to the FA-DMSO-EDC reaction mixture, stirred at 30°C in the dark overnight. The resultant mixture was dialyzed against 0.1M sodium phosphate buffer (pH 7.4), and changed PBS buffer every two hours for 3 days, followed by dialysis against water and changed water every 6 hours for 3 days using a dialysis bag (cut-off MWCO 7KDa). The synthesized product of folic acid-cholesterol-chitosan (FACC) was obtained by lyophilization.

### Characterization of FACC conjugates

The structure of chitosan, CH-CS and FACC was analyzed by Fourier transform infrared (FTIR) spectroscopy (WGH-30, Gang Dong Technology Co. Ltd., Tianjin, China) and ^1^H NMR spectrum (in D_2_O, 500 MHz, Bruker spectrometer, Switzerland). For IR spectrum analysis, about 5 mg of dried chitosan, or CH-CS or FACC sample was mixed with 50 mg of KBr and grounded with pestle and mortar to make thin discs, respectively. The spectra were scanned at room temperature from 400 to 4000 cm^−1^ wavelength with a resolution of 2 cm^−1^. For ^1^H NMR analysis, chitosan, CH-CS, and FACC were dissolved in deuterated reagents and confirmed by ^1^H NMR spectrometer.

### Critical micelle concentration (CMC)

The CMC of the FACC was determined by using pyrene (>95%, Sigma, St Louise, MO, USA) as a hydrophobic probe in fluorescence spectroscopy (Perkin Elmer, IS55, USA). The pyrene solutions (6.0×10^−5^ mM) in methanol were added into the test tubes and evaporated under a stream of nitrogen gas to remove the methanol. A serial of FACC solutions with different concentrations ranging from 2 to 250 μg/ml were prepared and then left to equilibrate with a constant pyrene concentration of 60 nM for 24h at 37°C. Fluorescence excitation spectra were measured at the excitation wavelength of 337 nm, and the emission wavelength was 350 nm – 450 nm for emission spectra. Both excitation and emission bandwidths were set at 8 nm. The peak height intensity ratio (I384/I372) of the third peak I384 to the first peak I372 was plotted against the logarithm of polymer concentration. The intersection of the tangent to the curve at the inflection with the horizontal tangent through the points at low polymer concentrations was taken as the CMC value.

### Preparation of PTX loaded FACC micelles

PTX loaded micelles were prepared with FACC by a dialysis method. Briefly, 10 mg FACC was dissolved in 5 ml of HCl-THF mixture consisted of 100 mM hydrochloric acid (HCl) and tetrahydrofuran (THF) (1:1, v/v) to make a FACC solution. Various concentrations of PTX were dissolved in methanol and added into FACC solution then sonicated with ultrasonic cleaner in 25°C water bath for 30 min with the power output of 200W. The blend solution (5 ml) was dialyzed against 500 ml de-ionized water and changed water every 2h using a dialysis bag (cut-off MWCO 7 kDa). Followed that, the PTX-FACC micelles were obtained by lyophilization and kept for further analysis.

### Characterization of PTX-FACC micelles

The particle sizes and polydispersities of PTX-FACC micelles diluted in de-ionized water were determined using dynamic light scattering system Zetasizer Nano ZS90 (Malvern Instruments, UK) in triplicate.

PTX-FACC micelles (1 mg) were dissolved with 1 ml of de-ionized water to make a testing sample. One-drop sample was placed on a carbon-coated film 300 mesh copper grid and allowed to sit for 5 min or until air-dried. The sample was stained with uranyl acetate (1%, W/V) for 5 min, and any excess uranyl acetate was removed with filter paper. Morphological characteristics of the micelles were examined using a high-resolution transmission electron microscope (TEM; JEM-2000EX, JEOL Co. Japan).

The encapsulation efficiency (EE) and loading efficiency (LE) of PTX-FACC micelles were determined by the high-performance liquid chromatography (HPLC, UV-230 II, Yilite, Dalian, China) assay described as follows: PTX-FACC micelles (1 mg) were dissolved in 2 ml of methanol and water mixture (80: 20, v/v), vortexed, then sonicated for 30 min. The PTX concentrations were analyzed by HypersilODS-C_18_ column (250 mm × 4.6 mm, 5 μm) using methanol: water (80: 20, v/v) as a mobile phase, the PTX was detected at the wavelength of 227 nm, the flow rate was 1.0 mL/min and the column was kept at 25°C. EE and LE were calculated by the following equations:

EE% = [weight of encapsulated drug/weight of total drug] ×100%

LE% = [weight of encapsulated drug/weight of total drug and vector] ×100%

### *In vitro* release of PTX-FACC micelles

The *in vitro* release behavior of PTX from PTX-FACC micelles were determined by dialysis method for up to 7 days. Each 50 mg PTX-FACC micelles (contains 5 mg PTX) were resuspended in 5 ml de-ionized water. This PTX-FACC micelle solution was dialyzed using a membrane dialysis bag (1ml/bag; cut-off MWCO 7 kDa) in 50 ml PBS at pH 5.0, 7.4 and 9.0 at 37±0.5°C with continuous stirring for 7 days, respectively. To meet the sink condition, 0.5 ml of polysorbate 80 was included in 50 ml of releasing medium as a solubilizer (1%, v/v). An anticancer drug, Taxol® (1 ml of 1 mgPTX) was also dialyzed in 50 ml PBS at pH 7.4 and used as control labeled PTX. At each predetermined time point, 2 ml releasing medium was taken, refilled with the same amount of the fresh medium. The concentration of the released PTX was determined by HPLC as described in the “Determination of encapsulation efficiency” section.

### Cytotoxicity assay

The cytotoxicity of PTX-FACC micelles on two type cells was tested by MTT method. Briefly, Hela (folate receptor-positive) and A549 (folate receptor-negative) cells were seeded at a density of 1×10^4^/well in 100 μl of medium in a 96-well culture plate and incubated for 24 hrs at 37°C. Different concentrations (0.1, 1, 10, 100 μg/ml) of PTX-FACC micelles and Taxol® were added to the cells and incubated for another 24 hrs at 37°C, respectively. Discard the culture medium and then wash the plate with PBS. MTT solution (0.5%, 10 μl) was added to the cells in each well and incubated for another 4 hrs at 37°C. Remove the culture medium, wash the plate with PBS and then added into 200 μl DMSO. The culture plate was incubated for 30 min at 37°C. Absorbance was detected on a Microplate Reader (Thermo Multiskan Ascent 354, USA) at 492 nm. The cell viability was calculated as the following formula: Viable cells% = (OD of treated group/OD of control group) × 100%. Different concentrations (1, 5, 10, 50, 100, 500 μg/ml) of FACC micelles were added to the two cells lines and treated as mentioned above.

### Intracellular uptake of PTX-FACC micelles

In order to test the distribution of FACC micelles in Hela and A549 cells, FITC-conjugated FACC micelles were created according to the published protocol [28]. Hela and A549 cells were seeded at a density of 5×10^5^/well in a 6-well plate and incubated for 24 hrs at 37°C. Then 20 μl of FITC-FACC micelles were added into each well and FITC solution only was used as control. After 2 h of incubation, all wells were incubated with rabbit monoclonal anti-human folate receptor (1:500) antibody for another 2 hrs at 37°C, followed by incubation with TRITC-labeled goat anti-rabbit immunoglobulin antibody (1:150) for 20 min. All wells were washed three times with PBS, and nucleus was stained for 10 min with Hoechst (50 μg/ml). The staining solution was removed and washed three times with PBS. The fluorescence in each sample was monitored by a SP5 laser scanning confocal microscopy (Germany).

### Statistical analysis

All experiments were repeated at least three times and the data were presented as the mean ± standard deviation (SD). Tukey's test was performed to determine statistical significance and a *P* value < 0.05 was considered to be significantly different.

## CONCLUSION

The novel amphiphilic folate acid-cholesterol-chitosan nanoscale micelles were prepared to the targeted delivery of paclitaxel. The PTX-FACC micelles developed in this study had suitable diameter, high encapsulation efficiency and loading capacity, uniform spherical morphology, and slow release *in vitro*. In addition, *in vitro* cytotoxicity assays confirmed that PTX-FACC micelles have a higher cytotoxicity than the Taxol®. PTX-FACC micelles may provide a potential tool to target FR-positive cancer cells and hence is a promising candidate for anticancer activity.
